# Supplementary material for: Cooling Performance Prediction of Particle-Based Radiative Cooling Film Considering Particle Size Distribution
Source: Micromachines (Basel). 2024 Feb 21;15(3):292. doi: 10.3390/mi15030292 (PMC10972170; doi:10.3390/mi15030292)
Supplement: Supplementary file 1 [file micromachines-15-00292-s001.zip › micromachines-2885029-supplementary.pdf]

1. Calculation of cooling power

In radiative cooling, cooling power is represented by the net cooling power (P_{net}), which accounts for all heat exchange processes. The net cooling power (P_{net}) is calculated using the following equation [1–3]:

$$P_{net}(T) = P_{rad}(T) - P_{atm}(T_{atm}) - P_{sun} - P_{cc} \tag{S1}$$

In Eq. (S1), T represents the surface temperature of the particle-based radiative cooling (PBRC) film. P_{rad} is the radiated power by the PBRC film. The emissive power by the PBRC film is calculated by integrating the spectral hemispherical emissive power, and it can be determined from Eq. (S2):

$$P_{rad}(T) = \int d\Omega cos\theta \int_0^\infty d\lambda I_{BB}(T,\lambda)\varepsilon(\lambda,\theta) \tag{S2}$$

where $I_{BB} = (2hc^2/\lambda^5)/[c^{hc/\lambda K_B T} - 1]$ represents the Blackbody radiation at T, and wavelength λ. $\varepsilon(\lambda,\theta)$ is the radiance as a function of wavelength λ and angle θ of the PBRC film. According to Kirchhoff's law, spectral emissivity replaces spectral absorptance [4]. Here, h, c, and k_B represent Planck's constant, the speed of light, and the Boltzmann constant, respectively. The atmospheric thermal radiation by the PBRC film (P_{atm}) can be represented as Eq. (S3):

$$P_{atm}(T_{atm}) = \int d\Omega cos\theta \int_0^\infty d\lambda I_{BB}(T_{atm},\lambda)\varepsilon(\lambda,\theta)\varepsilon_{atm}(\lambda,\theta) \tag{S3}$$

where $\varepsilon_{atm}(\lambda,\theta) = 1 - t(\lambda)^{1/cos(\theta)}$ represents the atmospheric radiation as a function of angle, and $t(\lambda)$ is the atmospheric transmittance in the zenith direction [3].

P_{sun} is the absorbed power of the incident solar energy, and it is calculated as equation Eq. (S4) :

$$P_{sun} = \int_0^\infty d\lambda \varepsilon(\lambda,\theta) I_{AM1.5}(\lambda) \tag{S4}$$

where the solar spectrum is denoted as $I_{AM1.5}$, using an air mass of 1.5.

$$P_{cc} = h_c(T_{atm} - T) \quad \text{(S5)}$$

In Eq. (S5), P_{cc} is the heat loss due to surrounding environment conduction and convection. This study has set the convection heat transfer coefficient h to 6 W/m^2·K [5]. The cooling performance of the PBRC film refers to the net cooling power when the temperature difference between the T and T_{atm} is zero.

2. The number of photons in the Monte-Carlo method

In this work, we utilized the Monte-Carlo method to calculate the spectral reflectance and absorptance. The Monte-Carlo method is a probabilistic simulation that assesses the radiative properties of particle-based radiative cooling (PBRC) structures by tracking photons. It involves probabilistic considerations of the transmission, reflection, and absorption events during photon interactions with the medium and the particles within it. To ensure accurate prediction of the radiative properties, the selection of an appropriate number of photons is crucial, balancing calculation accuracy and computation time efficiency.

To determine an appropriate number of photons, the cooling power was calculated 10 times according to the number of photons, as illustrated in Fig S1. The thickness of the PBRC film was fixed at 1 mm and the volume fraction was set to 20%. The number of photons was evaluated for three cases: 10^3, 10^4, and 10^5. The computation time for each case sequentially increased to approximately 10 minutes, 4 hours, and 11 hours. A lower number of photons resulted in faster computation time but lower consistency of the cooling power due to larger deviations. In contrast, increasing the number of photons slowed down the computation time but the deviation gradually decreases. After ten calculations for each case, the average cooling powers were calculated as 57.2, 58.1, and 58.1 W/m^2, respectively. Notably, the cooling power of 10^3 photon case decreased by 0.9 W/m^2 compared to cases with 10^4 and 10^5 photons. Mean Squared Error(MSE) was employed to assess accuracy based on the number of photons, resulting in decreasing MSE values of 3.3, 0.55, and 0.04. Lower MSE value indicates higher consistency and accuracy, emphasizing the importance of a higher number of photons. Although further reducing the error is possible with over 10^5 photons, the computation time can increase. Therefore, we selected a photon number of 10^5, balancing computation time, repeatability and accuracy of the radiative properties.

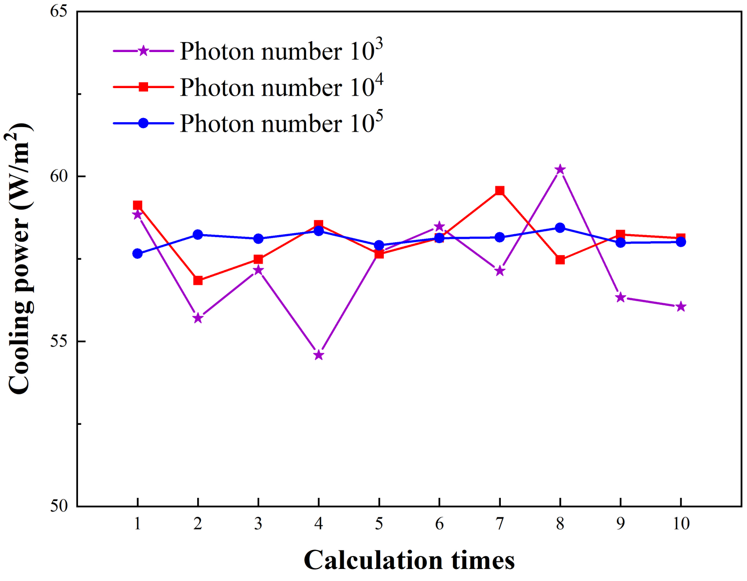

Figure S1. Cooling power calculated 10 times according to the number of photons

3. Rayleigh scattering

Rayleigh scattering occurs when light encounters with particles smaller than the wavelength regime. It depends on the size of the particles, and the scattered irradiance I_s by Rayleigh scattering is as follows:

$$I_s = \frac{8\pi^4 M a^6}{\lambda^4 r^2} \left| \frac{n^2 - 1}{n^2 + 2} \right|^2 (1 - cos^2\theta) I_i \tag{S6}$$

In Eq. (S6), M, a, n represent the number, radius, and refractive index of the particle, respectively. θ is the scattering angle, and I_i is the incident light. λ and r represent wavelength and the distance to particle. In the end, as the radiation irradiance is proportional to the sixth power of the radius of the particle, it can be seen that the scattering effect is negligible in the case of D_{min}, characterized by an extremely small particle size.

4. Circle detected image

In this study, We used 1 µm size of Al_2O_3 micro-nanoparticles(Ditto Technology, Korea). A total of 2,013 particles were detected from 12 SEM images using the Circle Hough Transform (CHT). Representative examples of the circle object detection (highlighted by red lines) are presented in Figure S2.

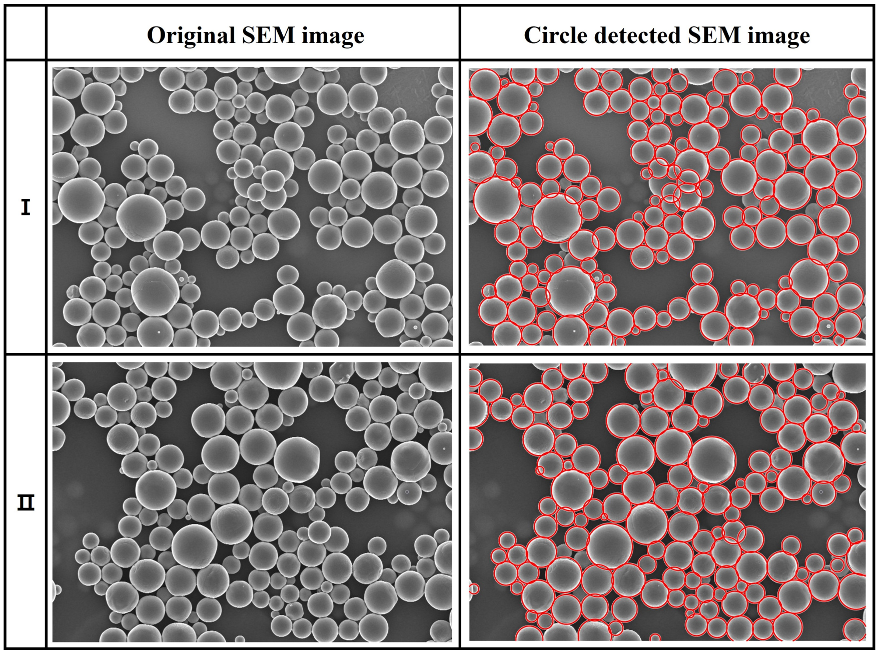

Figure S2. Original SEM image (Left) and circle detected image (Right) of Al_2O_3 micro-nanoparticles

5. Fabrication of PDMS/Al_2O_3 sample

Polydimethylsiloxane elastomer kit (PDMS, Sylgard 184), containing base and curing agent, was purchased from Dow Corning Co., Ltd. (Midland, MI, USA). Aluminum oxide (Al_2O_3, >99.5%, 1 µm) was purchased from Ditto Technology Co., Ltd. (Gunpo, Republic of Korea), and density of Al_2O_3 micro-nanoparticle is 3.95 g/cm^3. Silicone oil (SF1000N, 1 cSt) was obtained from KCC Silicone Corp. (Seoul, Republic of Korea).

To validate our proposed prediction method, a PDMS/Al_2O_3 sample was fabricated in six steps. 1) PDMS, curing agent, silicone oil, and Al_2O_3 micro-nanoparticles are mixed in the weight ratio (10:1:1:9.3). Silicone oil is utilized to uniformly disperse the Al_2O_3 micro-nanoparticles in the mixture due to high viscosity of PDMS. 2) The combined mixture is mixed for 30 minutes using a vortex mixer. 3) Ultrasonication is employed to disperse and mix the Al_2O_3 micro-nanoparticles evenly in the mixture, preventing agglomeration. 4) The

mixture is poured into a dish. 5) The gases are removed from the mixture using a vacuum chamber. 6) The mixture is cured at 55 °C for 12 hours.

References

1. Ma, Hongchen, et al. Flexible daytime radiative cooling enhanced by enabling three-phase composites with scattering interfaces between silica microspheres and hierarchical porous coatings. *ACS Appl. Mater. Interfaces* **2021**, *13*, 110561.
2. Lu, Xing, et al. Cooling potential and applications prospects of passive radiative cooling in buildings: The current state-of-the-art. *Renew. Sustain. Energy Rev.* **2016**, *65*, 1079–1097.
3. Raman, et al. Passive radiative cooling below ambient air temperature under direct sunlight. *Nature.* **2014**, *515*, 540–544.
4. Jaramillo-Fernandez, et al. Highly-scattering cellulose-based films for radiative cooling. *Adv. Sci.* **2022**, *9*, 2104758.
5. Liu, Yuting, et al. Acrylic membrane doped with Al2O3 nanoparticle resonators for zero-energy consuming radiative cooling. *Sol. Energy Mater Sol. Cells.* **2020**, *9*, 110561.
